# Atypical Presentation of Lobular Capillary Hemangioma in the Thoracic Spine: A Case Report

**DOI:** 10.7759/cureus.112163

**Published:** 2026-07-06

**Authors:** Kent W Sabatose, Daniel Wiggans, Wissam Elfallal

**Affiliations:** 1 Neurological Surgery, Lake Erie College of Osteopathic Medicine, Bradenton, USA; 2 Neurosurgery, NYU Langone Health, New York, USA; 3 Neurosurgery, AdventHealth Daytona Beach, Daytona, USA

**Keywords:** intradural extramedullary spine tumors, lobular capillary hemangioma, pyogenic granuloma, thoracic myelopathy, thoracic spine surgeries

## Abstract

Pyogenic granuloma, or lobular capillary hemangioma (LCH), is a vascular lesion that rarely occurs within the neuraxis and can lead to neurological deficits due to mass effect on neural structures. We present a 62-year-old man with LCH located in the intradural intramedullary region of the thoracic spine, treated surgically via gross total en bloc microsurgical resection without radiation or chemotherapy. No recurrence of LCH was noted at the 27-month follow-up, with near-complete restoration of neurological function. Our case supports that gross total resection alone of intradural intramedullary LCH can achieve favorable long-term clinical outcomes.

## Introduction

Pyogenic granuloma, also known as lobular capillary hemangioma (LCH), is a benign vascular lesion most commonly arising from the skin or oral mucous membranes [1,2]. LCH demonstrates a male predominance and typically presents as a red polypoid lesion with frequent bleeding and ulceration, occurring most commonly from childhood through the late twenties in men and between the third and fourth decades of life in women [1]. Atypical locations for LCH have been documented within the gastrointestinal tract, where lesions may mimic polyps, as well as intravascularly, most commonly involving the veins of the neck or upper extremities [3-5].

Risk factors associated with dermatologic LCH include trauma, hormonal changes related to pregnancy or oral contraceptive use, medications such as oral retinoids, immunosuppressants, and protease inhibitors, arteriovenous malformations, *Staphylococcus aureus* infection, and syndromes including neurofibromatosis type 1 and von Hippel-Lindau disease [6]. Viral oncogenes have also been proposed as a possible etiology of LCH, although this relationship remains controversial [6,7].

The primary treatment for LCH is surgical excision; however, medical therapies, including imiquimod, epidermal growth factor receptor inhibitors, and laser therapy, have also been described [8-10]. Despite the variety of proposed treatment strategies, no universally accepted standard of care exists because of limited prospective clinical data evaluating treatment efficacy [2,8].

LCH involving the neuraxis is exceedingly rare and most commonly occurs at the level of the conus medullaris or along the nerve roots of the cauda equina [11]. Intradural lesions are typically treated surgically, with en bloc resection preferred to reduce the bleeding risk associated with the lesion's high vascularity [12,13]. The roles of embolization, radiation therapy, and targeted antiangiogenic therapies remain poorly defined in the literature but may provide adjunctive therapeutic benefit in select cases [12,13].

Our case of intradural intramedullary LCH within the thoracic spine represents an extraordinarily rare presentation, with no previously reported cases in the literature to our knowledge. Gross total surgical resection without adjuvant radiation or chemotherapy resulted in significant neurological improvement with long-term remission. Given the rarity of neuraxial LCH and its uncommon thoracic spinal location, this case may help guide future diagnostic and therapeutic approaches for similar presentations.

## Case presentation

A 62-year-old man presented to the emergency department in September 2023 with a one-month history of progressively worsening thoracic back pain associated with gait instability, frequent falls, bilateral lower-extremity weakness, and progressive paresthesias. Numbness reportedly began in the left foot and gradually ascended bilaterally to a sensory level above the umbilicus. The patient additionally endorsed perineal numbness accompanied by bowel and bladder incontinence. A lumbar MRI obtained approximately one month before presentation was reportedly unremarkable.

Medical history was significant only for hyperlipidemia, and he had no prior surgical history. He denied tobacco, alcohol, or recreational drug use. Home medications included acetic acid-hydrocortisone otic solution, albuterol, atorvastatin, cyanocobalamin, ergocalciferol, folic acid, gabapentin, meloxicam, montelukast, and tizanidine. Initial vital signs were within normal limits.

Neurologic examination demonstrated an awake and fully oriented patient without cranial nerve deficits. Extraocular movements were intact without nystagmus or strabismus. Motor strength in the bilateral upper extremities was 5/5 throughout. Examination of the lower extremities demonstrated symmetric weakness graded 3/5 across the L2-S1 myotomes bilaterally. Sensory examination revealed a T8 sensory level with diminished light-touch and pinprick sensation, greater on the left than on the right. Proprioception was absent in both lower extremities. Saddle anesthesia and diminished rectal tone were present. Deep tendon reflexes were diminished in the lower extremities, while Hoffmann and Babinski signs were absent bilaterally.

**Figure 1:**
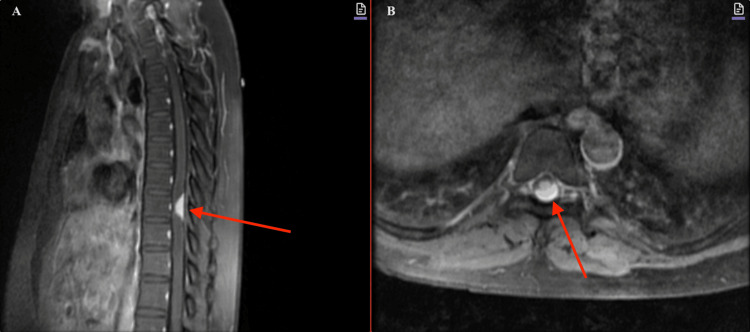
Preoperative T1-weighted contrast-enhanced MRI of the thoracic spine Sagittal (A) and axial (B) T1-weighted contrast-enhanced MRI images showing an intradural contrast-enhancing lesion centered at the T9 level (red arrows), causing severe spinal canal stenosis with spinal cord compression and edema.

Laboratory studies were unremarkable. MRI of the thoracic spine with and without contrast demonstrated a 2.3 × 10.2 × 22.4 mm contrast-enhancing intradural, suspected extramedullary lesion at the T9 level with severe anterior displacement and compression of the spinal cord. Extensive T2 hyperintensity within the thoracic cord was present, consistent with spinal cord edema/myelopathy (Figure 1). Differential diagnoses at this time included meningioma, nerve sheath tumor, and, less likely, metastatic disease because of the homogeneously enhancing lesion.

Given the patient's progressive neurologic decline and significant spinal cord compression, urgent surgical intervention was recommended. The patient was started on dexamethasone 10 mg every six hours and underwent T8-T10 laminectomy with microsurgical resection of the intradural intramedullary spinal tumor the following day.

Intraoperatively, the lesion was localized using intraoperative ultrasound with a hockey-stick probe. Following durotomy, the tumor was identified within the intradural intramedullary compartment. Significant arachnoid adhesions and thickening were encountered; however, a clear arachnoid plane allowed microsurgical dissection of the lesion from the surrounding neural structures without evidence of dural attachment. The lesion was internally decompressed and circumferentially dissected using microsurgical technique with microdissectors and Rhoton instruments, allowing gross-total en bloc resection. Frozen pathology was initially suggestive of hemangioblastoma. The patient tolerated the procedure well without intraoperative complications and was admitted postoperatively to the intensive care unit for hemodynamic monitoring and vasopressor support.

**Figure 2:**
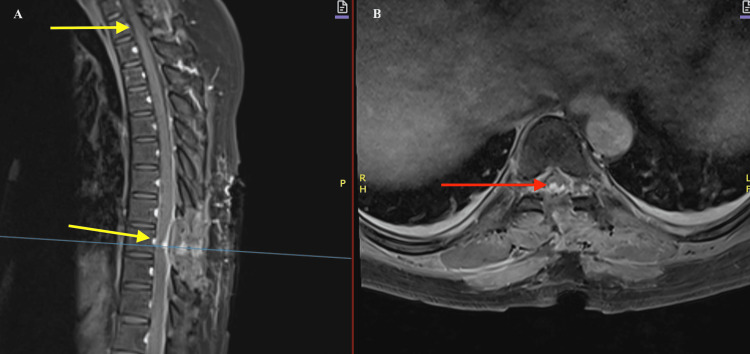
Postoperative day 0 contrast-enhanced MRI of the thoracic spine Sagittal (A) and axial (B) contrast-enhanced MRI images show a small residual focal area of enhancement within the spinal cord, to the right of midline and extending along the posterior aspect of the cord (red arrow), representing residual tumor versus postoperative changes. Residual spinal cord edema extending from the inferior endplate of T9 to the mid-T1 vertebral body (yellow arrows) is unchanged compared with Figure 1.

Postoperative MRI of the thoracic spine demonstrated expected postsurgical changes related to T8-T10 laminectomy and gross-total resection of the enhancing lesion. Minimal residual enhancement along the posterior right aspect of the cord was favored to represent postoperative change, although a small residual tumor could not be definitively excluded. Persistent but stable thoracic cord edema extending from T1 to T9 remained evident (Figure 2).

The patient was discharged on postoperative day 7 without complications. Final histopathology demonstrated LCH without evidence of malignancy. Immunohistochemical staining was negative for CD10, S100, PAX8, inhibin A, and TFE3. No radiation or chemotherapy was recommended for further treatment.

At the two-week follow-up, the patient reported marked neurologic improvement, including almost complete resolution of truncal numbness and substantial improvement in lower-extremity sensation and strength. Bowel and bladder dysfunction had completely resolved, and orthostatic symptoms were no longer present. On examination, strength was 5/5 throughout the bilateral upper extremities and right lower extremity. Strength in the left lower extremity was 5/5 in hip flexion and knee flexion/extension, with residual 4/5 weakness in ankle dorsiflexion, plantarflexion, inversion, and eversion. Mild proprioceptive deficits persisted. Reflexes were graded 2+ in the upper extremities and 1+ at the patellae bilaterally without clonus.

**Figure 3:**
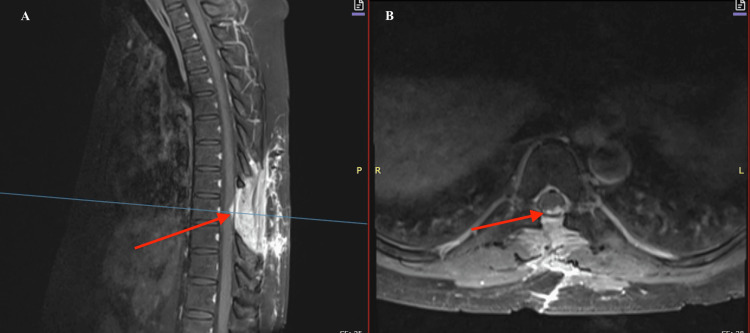
Three-month postoperative T1-weighted contrast-enhanced MRI of the thoracic spine Sagittal (A) and axial (B) T1-weighted contrast-enhanced MRI images showing postsurgical changes following T8 and T9 laminectomy and resection of the spinal cord tumor. Residual enhancement is present within the dorsal spinal cord at T9 (red arrows), measuring 0.5 × 0.8 × 1.3 cm.

At the three-month follow-up, the patient reported only minimal residual numbness involving the distal left foot and otherwise described a near-complete return to baseline neurologic function. Strength was 5/5 throughout all extremities, and axial thoracic pain had resolved. Follow-up MRI demonstrated complete resolution of the previously noted thoracic cord T2 hyperintensity without evidence of a recurrent enhancing lesion (Figure 3). Surveillance imaging at six-month intervals followed by annual imaging thereafter was recommended, and the role of stereotactic radiosurgery in the event of recurrence was discussed.

**Figure 4:**
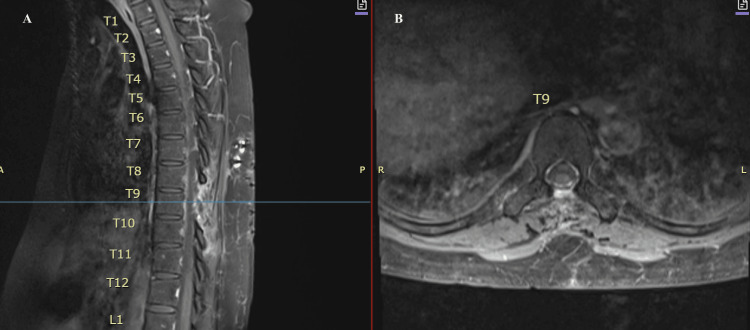
Nine-month postoperative T1-weighted contrast-enhanced MRI of the thoracic spine Sagittal (A) and axial (B) T1-weighted contrast-enhanced MRI images showing resolution of the previously observed small focus of dorsal spinal cord edema at T9. Mild spinal cord volume loss is present at the T9 level. Enhancement along the dorsal thecal sac at T9 is decreased, indicating probable postoperative improvement.

At the nine-month follow-up, MRI of the thoracic spine demonstrated continued improvement in postoperative appearance without evidence of residual or recurrent disease. Clinically, the patient remained neurologically intact and asymptomatic. He had returned to work and was ambulating independently without assistive devices. Bowel and bladder function had normalized completely, and he denied residual back pain. Neurologic examination demonstrated full strength throughout the bilateral upper and lower extremities with stable reflexes. Overall, the patient experienced near-complete neurologic recovery with no radiographic evidence of recurrence at nine months postoperatively (Figure 4).

Follow-up thoracic MRI with contrast at 15 months and 27 months continued to show resolution of enhancement along the dorsal thecal sac at T9 without evidence of recurrence. Clinically, the patient continued to demonstrate improvement on neurologic examination. He reported no back pain, paresthesias, weakness in his legs, or bowel/bladder incontinence. Overall, he rated himself as >90% improved from his preoperative baseline.

## Discussion

LCH involving the neuraxis is rare, particularly when located outside the conus medullaris or cauda equina, as demonstrated in our case [14]. In a case series of 18 patients with neuraxial LCH, 77.8% of lesions occurred within the thoracic spine, similar to our patient's presentation [15]. Most reported cases describe intradural extramedullary lesions, accounting for approximately 70% of neuraxial LCHs, whereas intramedullary lesions comprise approximately 14% of reported cases [16,17]. Our case represents an intradural intramedullary thoracic spinal LCH, consistent with the less commonly reported intramedullary neuraxial presentation. Most neuraxial LCHs occur between 40 and 60 years of age, although approximately 13 pediatric cases have been reported [18].

Symptoms of neuraxial LCH are primarily related to mass effect and commonly include pain, myelopathy, radiculopathy, paresthesias, and bowel or bladder dysfunction. Acute presentations secondary to intratumoral hemorrhage have also been reported [19]. Our patient presented subacutely with progressive myelopathy and bowel and bladder incontinence. The current standard of treatment is complete en bloc surgical resection, which is associated with low recurrence rates and favorable neurologic outcomes [20,21]. These lesions are often encapsulated, allowing identification of a distinct plane between the lesion and adjacent neural structures, as was noted in our case [11]. Complete en bloc resection was achieved in our patient, resulting in near-complete neurologic recovery without evidence of recurrence at 27 months postoperatively. In cases where gross-total resection is not feasible, spontaneous regression following subtotal resection has been reported, potentially secondary to reduced angiogenic signaling [22]. Additionally, radiotherapy may represent a reasonable adjunctive treatment option for cases unsuitable for total resection [22].

MRI characteristics of LCH typically include a well-circumscribed lesion that is isointense relative to the spinal cord on T1-weighted imaging, hyperintense on T2-weighted imaging, and demonstrates homogeneous contrast enhancement [23-25]. Histologically, neuraxial LCH resembles cutaneous LCH and demonstrates a lobular architecture composed of tightly packed capillary-sized vessels with expression of vascular endothelial growth factor in immature regions and absence of erythrocyte-type glucose transporter protein staining [26].

The rapid progression of our patient's neurologic symptoms over only one month, despite reportedly normal prior lumbar imaging, highlights the potential for rapid lesion growth and neurologic decline even in the absence of intratumoral hemorrhage [27]. To our knowledge, our case represents the first successful en bloc resection of an intradural intramedullary LCH without adjuvant radiation therapy, with radiographically confirmed nonrecurrence at >2 years postoperatively.

The retrospective nature and small sample size are inherent limitations of this study. Larger prospective clinical trials assessing total versus incomplete resection, as well as the addition of adjuvant therapies, including radiation and targeted chemotherapies, may help elucidate best practices for LCH that may be unresponsive to surgical management. Future directions for the treatment of surgically resistant LCH may include administration of interferon-alpha, which may improve postoperative outcomes in selected patients in whom the benefits of interferon-alpha administration outweigh the risks [28].

## Conclusions

Intradural intramedullary LCH involving the thoracic spine is exceptionally rare and may present with rapidly progressive myelopathy and severe neurologic dysfunction. Mass effect and hemorrhage pose a significant risk to neural structures due to the potential for rapid growth or expansion of LCH. Our case highlights the importance of including LCH in the differential diagnosis of avidly, homogeneously enhancing intradural lesions on MRI, particularly in patients presenting with acute or subacute neurologic decline. Gross-total en bloc microsurgical resection alone resulted in near-complete neurologic recovery without evidence of recurrence at the 27-month follow-up. Although additional cases are needed to better define long-term management, this report supports gross-total surgical resection as the primary treatment strategy for neuraxial LCH, with adjuvant therapy reserved for select circumstances.
